# The impact of the interaction between BDNF rs7103411 gene polymorphism and social activities on mild cognitive impairment in community-dwelling elderly adults

**DOI:** 10.3389/fpsyt.2024.1469671

**Published:** 2025-01-14

**Authors:** Zhenkun Tan, Junjiao Ping, Ying Zhang, Chuijia Kong, Jiali Luo, Xinxia Liu

**Affiliations:** Department of Psychiatry, Third People’s Hospital of Zhongshan City, Zhongshan, China

**Keywords:** gene polymorphism, social activities, MCI, BDNF, elderly adults

## Abstract

**Objective:**

To investigate the correlation between BDNF gene polymorphism, BDNF levels, and susceptibility to mild cognitive impairment (MCI).

**Methods:**

In this study, we investigated 107 elderly adults individuals from a community in Zhongshan, Guangdong Province, with an average age of 73.17 ± 7.081 years. The participants included 52 patients with Mild Cognitive Impairment due to Alzheimer’s Disease and 55 cognitively normal elderly adults control subjects. The two groups were matched based on gender, age, and education level. We assessed their cognitive functions and analyzed their genotypes and serum BDNF levels. Analysis of covariance (ANCOVA) was used to evaluate the differences in serum BDNF levels between the MCI group and the control group. Multivariate linear regression was utilized to analyze the association between BDNF levels and susceptibility to MCI, as well as cognitive functions. Multivariate logistic regression was employed to investigate the association between BDNF gene polymorphisms and the risk of developing MCI, along with their interactions.

**Results:**

The ANCOVA analysis indicated that there was no significant difference in serum BDNF levels between the MCI group and the control group (*P* > 0.05). Correlation analysis revealed a negative correlation between Mini-Mental Status Examination (MMSE) total scores and MCI (*r* = -0.461, *P* = 0.001), with significant correlations observed in orientation (*r* = -0.420, *P* = 0.002). Multiple linear regression analysis showed that specific polymorphisms, including rs7103411 (CT+TT vs. CC), rs6265 (CT and CT+TT vs. CC), rs11030104 (AG and AG+GG vs. AA), and rs988748 (CG+CC vs. GG), were significantly associated with decreased serum BDNF levels (*P* < 0.05). Multivariate logistic regression showed that rs7103411 polymorphism was associated with susceptibility to MCI; individuals with the CT or CC genotype had a 0.370 times lower risk of developing MCI compared to those with the TT genotype (*OR* = 0.370, 95% *CI*: 0.141-0.970, *P* = 0.043). A significant interaction was found between rs7103411 and social activity, which influenced the risk of developing MCI. Specifically, individuals with the CT or TT genotype of rs7103411 who engaged in social activities had a significantly lower risk of developing MCI (*OR* = 0.32, 95% *CI*: 0.117-0.878, *P* = 0.027).

**Conclusion:**

This study indicates that BDNF rs7103411、rs6265、rs11030104 and rs988748 are associated with decreased serum BDNF levels in MCI patients. Individuals carrying the TT genotype in the BDNF rs7103411 gene are associated with an increased susceptibility to MCI. Individuals with the rs7103411 CT or TT genotype who participated in social activities showed a significantly reduced risk of developing MCI, suggesting that the interaction between the BDNF rs7103411 genotype and social activity can help reduce the risk of MCI.

## 1 Introduction

Mild Cognitive Impairment (MCI) is one of the common neurocognitive disorders and a significant public health issue, with its prevalence increasing with age. Its prevalence increases with age, the prevalence is 6.7% in individuals aged 60-64 years and 25.2% in those aged 80-84 years (1). MCI due to AD is considered to be associated with an increased risk of developing dementia, particularly Alzheimer’s dementia (1). Therefore, it is essential to identify and intervene early in cases of MCI (1). A nationwide cross-sectional study in China showed that the prevalence of mild cognitive impairment (MCI) is 15.5%, with approximately 38.77 million people affected by this condition (2). Early intervention can help delay the progression of the disease and ensure better outcomes.

Brain-Derived Neurotrophic Factor (BDNF) is a member of the neurotrophin family that plays an important role in promoting the growth, differentiation, and survival of neurons. It is crucial for neuroplasticity and cognitive function (3). Some studies suggest that BDNF levels are linked to the occurrence of neurodegenerative disorders such as Alzheimer’s Disease (AD), Frontotemporal Dementia (FTD), Lewy Body Dementia (LBD), and Vascular Dementia (VAD). However, studies on peripheral BDNF levels in patients with AD and MCI have yielded inconsistent results. On one hand, some studies have found significantly lower serum BDNF levels in AD and MCI patients compared to healthy controls (4–8). On the other hand, other studies have reported increased peripheral levels of BDNF in AD and MCI patients compared to healthy individuals (9–11). Furthermore, there are discrepancies in the trends of BDNF level changes: some studies show a decrease in peripheral BDNF levels during the MCI stage and an increase during the AD stage (12), while others indicate an increase during the MCI stage followed by a decrease during the AD stage (13). These conflicting results may be related to sample differences (population, medication use, etc.), methodological differences (cognitive assessment tools, sample handling, etc.), and environmental factors (lifestyle, etc.).

BDNF gene polymorphism is associated with neurodegenerative diseases, with the rs6265 (Val66Met) polymorphism linked to the risk of Alzheimer’s Disease (AD) (14, 15) and related to cognitive function (6, 16–21). Moreover, the secretion of BDNF is affected by BDNF rs6265 polymorphism (22, 23), indicating that BDNF gene polymorphism may impact the expression and activity of the protein, thereby influencing individual neuroplasticity and cognitive function. A meta-analysis indicated that individuals carrying the Met/Met genotype not only have poorer cognitive abilities but also lower serum BDNF levels (19). Studies show that BDNF gene polymorphism is significantly associated with memory function (24), while other studies find no significant link between gene polymorphism and cognitive function (25). These conflicting results may be due to the complex interactions between multiple genes affecting cognitive function and insufficient consideration of gene-environment interactions (26, 27). Although previous research has focused on the relationship between BDNF gene polymorphisms and cognitive functions, the majority of studies have concentrated on rs6265. There is less research on other polymorphic segments, particularly concerning interactions with environmental factors such as lifestyle.

The impact of genetics and environment on Alzheimer’s Disease (AD) is not independent; the interplay between immutable risk factors such as genetics and modifiable factors like environment and physical activity can influence the onset of AD (28). Besides the impact of BDNF gene polymorphism on cognition, social activity, as a significant lifestyle factor, has been shown to positively affect cognition in the elderly adults (29, 30). Active participation in social activities not only provides cognitive stimulation but may also indirectly affect BDNF levels and function by reducing stress and enhancing social support (31). Engaging more in social activities could help prevent or delay cognitive decline in elderly adults patients with Mild Cognitive Impairment (MCI) (32). A longitudinal study showed that in elderly individuals without cognitive impairment potentially modifiable factor such as social isolation may contribure to a condition of biopsychosocial frailty, which may increase the risk of dementia (33). Therefore, exploring the interactions between BDNF gene polymorphism, BDNF levels, and social engagement is crucial for understanding the complex etiology of MCI and its prevention strategies.

This study aims to explore the relationship between BDNF gene polymorphism and susceptibility to MCI, as well as the connection between BDNF levels and cognitive function in MCI patients. Additionally, the study will examine the impact of the interaction between social activities and BDNF polymorphism on the risk of MCI. Due to the lack of obvious symptoms, elderly individuals with mild cognitive impairment (MCI) may not receive timely diagnosis and medical treatment, leading them to often reside in the community rather than in hospitals. Therefore, early identification of MCI among community-dwelling elderly individuals is crucial. This study focuses on the elderly community population, aiming to provide a theoretical basis for the early diagnosis and personalized intervention of MCI.

## 2 Methods

### 2.1 Study sampling

In May 2023, a simple random sampling was employed to select one district in Zhongshan, Guangdong Province. A total of 500 community-dwelling elderly persons aged 60 and over were randomly selected to participate in the questionnaire survey. During the screening process, neuropsychological tests, including the Mini-Mental Status Examination (MMSE), were used to assess cognitive function. The inclusion criteria encompassed individuals aged 60 years and above who did not have any known major cerebrovascular diseases, dementia or other pathological cognitive impairments, acute functional mental disorders (including schizophrenia or bipolar disorder), stroke history, etc. Of the 91 people diagnosed with MCI, 52 agreed to participate in the study. A total of 55 participants were matched by sex, age, and education as a control group to the cognitively normal people in the same area. A total of 107 people were actively involved in the study by undergoing blood collection procedures.

### 2.2 Clinical assessment

Mini-Mental Status Examination(MMSE) is currently the most valuable screening tool for identifying the early stages of Alzheimer’s disease. It is widely used in community epidemiological surveys and has good reliability and validity. In this study, MMSE was used to screen for Mild Cognitive Impairment (MCI) by assessing various aspects of cognitive function, including orientation, memory, attention and calculation, recall ability, and language ability. The scale is composed of 30 items, including orientation, memory, attention and calculation, recall ability, and language ability in 5 dimensions; the correct answer gets 1 point, and the wrong answer gets 0 points, for a total of 30 points. Activities of daily living (ADL)(Wade and Collin, 1988) scale to measure the daily living ability of community-dwelling elders. This scale comprises ten items, including the ability to: eat, bathing, grooming, dressing, stool control, urination control, toileting, transfer, walking on level ground, and going up and downstairs. Responses to each of the ten items on the scale are scored as 0 (cannot perform or can partially perform) or 5 (can perform). The total score ranges from 0 (low-functioning, dependent) to 100 (high-functioning, independent). Collection of Demographic Characteristics: The demographic characteristics of participants were collected through a standardized questionnaire, including information such as age, gender, education level, and lifestyle. The questionnaire was completed during face-to-face interviews conducted by trained investigators to ensure data accuracy and consistency. Height and weight were measured on-site by medical personnel.

Diagnostic criteria for MCI due to AD (34): (1) Concern regarding a change in cognition; (2) Impairment in one or more cognitive domains; (3) Preservation of independence in functional abilities; (4) Not demented.The inclusion criteria encompassed individuals aged 60 years and above who did not have any known major cerebrovascular diseases, dementia or other pathological cognitive impairments, acute functional mental disorders (including schizophrenia or bipolar disorder), stroke history and traumatic brain injury. Patients with a history of alcohol abuse, drug addiction, and long-term use of medications that affect cognitive function, such as glucocorticoids, antipsychotic drugs, and sedative-hypnotic drugs, are excluded.

### 2.3 Brain-derived neurotrophic factor

Serum BDNF levels in MCI patients and control groups were measured using the enzyme-linked immunosorbent assay (ELISA) calibrated with an ELISA reader. The ELISA kits were purchased from BOSTER, and the experiment was conducted strictly following the instructions provided with the kits. The equipment used included a centrifuge (XinAo, GENIUS 6K-C model), a full-wavelength ELISA reader (ThermoFisher, Multiskan SkyHigh model), a constant temperature water bath (BIOBASE, BJPX-WB26 model), and a washer (Antu, IWO-960 model). Blood samples were collected after obtaining written informed consent from the participants, who voluntarily joined the study following a clear explanation of its purpose and procedures. The blood collection was conducted by certified medical professionals under aseptic conditions, ensuring the validity of the samples and the safety of the participants. Furthermore, all research procedures were approved by an ethics committee, adhering to international ethical standards.

### 2.4 Genotyping

Peripheral venous blood was collected into an ethylenediaminetetraacetic acid-containing vacuum anticoagulant tube and centrifuged at 3,300 rpm at room temperature. The leukocytes in the middle layer were collected and stored in a cryopreservation tube at −80°C for subsequent use. A TIANamp Genomic DNA Kit (centrifugal column type; DP304; Tiangen, Beijing, China) was used to extract genomic DNA, and the optical density was determined using a spectrophotometer (Thermo Fisher Scientific, Waltham, MA, United States). Genomic DNA extraction results were evaluated by gel electrophoresis. By analyzing the two key loci of the APOE gene, the 112th position (rs429358) and the 158th position (rs7412), the genotype is determined. The ϵ2, ϵ3, and ϵ4 alleles are defined by the base combinations at these loci: T at the 112th position and T at the 158th position indicates ϵ2; T at the 112th position and C at the 158th position indicates ϵ3; and C at both the 112th and 158th positions indicates ϵ4. If the genotype contains the ϵ4 allele (e.g., ϵ3/ϵ4 or ϵ4/ϵ4), the individual is classified as an APOE4 carrier. In this study, genetic polymorphisms related to cognitive function (22) (e.g., rs6265, rs7103411), BDNF expression (35) (rs11030100), Alzheimer’s disease (36) (rs11030104), and other psychiatric disorders (37) (rs988748) were selected to investigate their potential associations in patients with mild cognitive impairment (MCI).

The results of linkage disequilibrium analysis show that rs11030104 and rs6265 (R²=0.907, DP=0.961), rs11030104 and rs7103411 (R²=0.927, DP=1), rs11030104 and rs988748 (R²=0.908, DP=1), rs6265 and rs7103411 (R²=0.837, DP=0.960), rs6265 and rs988748 (R²=0.817, DP=0.960), and rs7103411 and rs988748 (R²=0.942, DP=0.981). Among these, the DP values for rs11030104, rs7103411, and rs988748 are equal to 1, indicating that these three SNPs are in complete linkage. Hardy-Weinberg analysis shows that all loci, except for rs11030100 (*P <*0.001), follow Hardy-Weinberg equilibrium.

### 2.5 Statistical analysis

Analysis was conducted using R software version 4.1.3. Normally distributed continuous variables were described as mean ± standard deviation, and non-normally distributed variables were described using the median (interquartile range). Categorical variables were expressed as percentages (%). The t-test and chi-square test were used to assess differences between the two groups. Analysis of covariance (ANCOVA) was used to compare differences in BDNF levels between the MCI group and the control group. Multiple linear regression was used to analyze the association between BDNF gene polymorphism and serum BDNF levels. Multivariate logistic regression was used to assess the association between BDNF polymorphism and susceptibility to MCI, as well as the interaction between the BDNF gene and environmental factors.

## 3 Results

### 3.1 Basic characteristics of the study subjects

The study included 107 participants, consisting of 52 MCI patients and 55 cognitively normal elderly adults individuals. The basic characteristics of the subjects are presented in **Table 1**. There were no statistically significant differences between the MCI group and the healthy control (HC) group in terms of age, gender, BMI, education level, social activities, and serum BDNF concentrations (all *P* > 0.05).

**Table 1:** Basic characteristics of MCI and HC [n (%)].

	HC (N=55)	MCI (N=52)	Overall (N=107)	*χ*²/*t*	*P*
Age,Mean ± SD	73.33 ± 6.861	72.98 ± 7.382	73.17 ± 7.081	0.252	0.802
Sex, (n, %)				2.688	0.101
Male	17 (30.9)	9 (17.3)	26 (24.3)		
Female	38 (69.1)	43 (82.7)	81 (75.7)		
BMI	24.03 ± 3.151	25.40 ± 6.993	24.62 ± 5.356	-1.313	0.192
Education, (n, %)				1.612	0.657
Illiterate	12 (21.8)	17 (32.7)	29 (27.1)		
Primary school	18 (32.7)	15 (28.8)	33 (30.8)		
Junior high school	16 (29.1)	13 (25.0)	29 (27.1)		
Senior high school and above	9 (16.4)	7 (13.5)	16 (15.0)		
Social Activity, (n, %)				0.674	0.412
Participate	38 (69.1)	32 (61.5)	70 (65.4)		
Do Not Participate	17 (30.9)	20 (38.5)	37 (34.6)		
BDNF,ng/mL,Mean ± SD	9.91 ± 3.431	9.43 ± 2.570	9.67 ± 3.038	0.821	0.413
APOE-4 Carrier, (n, %)				–	0.618
+	3 (5.5)	1 (2.0)	4 (3.7)		
–	52 (94.5)	51 (98.0)	103 (96.2)		

### 3.2 Correlation between serum BDNF levels and cognitive function

As shown in **Table 2**, in all participants, including both the MCI group and the healthy control group, BDNF levels were not significantly correlated with MMSE total scores or scores in various cognitive domains (all *P* > 0.05). In the normal control group, BDNF levels were also not significantly correlated with MMSE total scores or scores in any cognitive domains (all *P* > 0.05). However, for MCI patients, BDNF levels were significantly correlated with MMSE total scores (*r* = -0.461, *P* = 0.001) and orientation (*r* = -0.420, *P* = 0.002), but not with memory, attention and calculation, language abilities, or recall capabilities (all *P* > 0.05).

**Table 2:** Correlation between BDNF levels and cognitive performance measures.

	Overall	MCI	HC
Orientation Ability	-0.189 (0.052)	**-0.420 (0.002)**	-0.128 (0.352)
Memory Ability	-0.099 (0.309)	-0.259 (0.064)	-0.017 (0.901)
Attention And Computational Ability	-0.018 (0.857)	-0.101 (0.475)	-0.018 (0.896)
Linguistic Ability	-0.158 (0.103)	-0.253 (0.071)	-0.175 (0.202)
Recall Ability	-0.031 (0.751)	-0.172 (0.233)	-0.024 (0.862)
MMSE Score	-0.156 (0.109)	**-0.461 (0.001)**	-0.074 (0.592)

*Values shown as r(*P*); Bold values indicate P < 0.05.

### 3.3 BDNF gene polymorphism and susceptibility to MCI

A comparison of genotype frequencies between MCI patients and the control group is presented in **Table 3**. After adjusting for age, gender, educational level, and APOE carrier status, the genetic model analysis of the BDNF rs7103411 locus between the MCI group and the HC group showed that compared to the frequency of CT plus CC genotypes, the frequency of the TT genotype was significantly higher in the MCI group compared to the HC group, indicating that the TT genotype may be associated with an increased risk of developing MCI, although the significance was modest (*P* = 0.043). No statistically significant differences were found at other genetic loci. (all *P >*0.05).

**Table 3:** Comparison of genotype frequencies between MCI and control groups.

Gene Locus	Genotyping	MCI (n, %)	HC (n, %)	*OR (95% CI)*	*P*
BDNF rs7103411	TT	17 (32.7)	9 (16.4)	1.000 (Reference)	
	CC	11 (21.2)	14 (25.5)	0.358 (0.112-1.151)	0.085
	CT	24 (46.2)	32 (58.2)	0.375 (0.136-1.034)	0.058
	CT+CC vs. TT	35 (67.3)	46 (83.6)	0.370 (0.141-0.970)	**0.043**
	CT+TT vs. CC	41 (78.8)	41 (74.5)	3.691 (0.343-39.701)	0.281
BDNF rs6265	CC	13 (24.1)	12 (23.1)	1.000 (Reference)	
	TT	11 (20.4)	17 (32.7)	1.764 (0.569-5.464)	0.325
	CT	30 (55.6)	23 (44.2)	0.857 (0.320-2.297)	0.759
	CT+TT vs. CC	40 (76.9)	41 (75.9)	1.097 (0.434-2.774)	0.845
	CT+CC vs. TT	35 (67.3)	43 (79.6)	1.958 (0.786-4.877)	0.149
BDNF rs11030104	AA	11 (21.2)	13 (23.6)	1.000 (Reference)	
	GG	18 (34.6)	10 (18.2)	2.202 (0.695-6.979)	0.180
	AG	23 (44.2)	32 (58.2)	0.827 (0.306-2.234)	0.708
	AG+GG vs. AA	41 (78.8)	42 (76.4)	1.144 (0.449-2.917)	0.777
	AG+AA vs. GG	34 (65.4)	45 (81.8)	2.509 (0.989-6.367)	0.053
BDNF rs11030100	GG	4 (7.7)	6 (12.2)	1.000 (Reference)	
	TT	44 (84.6)	41 (83.7)	0.672 (0.171-2.644)	0.570
	GT	4 (7.7)	2 (4.1)	0.379 (0.044-3.243)	0.376
	GT+TT vs. GG	43 (87.8)	48 (92.3)	0.645 (0.165-2.524)	0.529
	GT+GG vs. TT	8 (16.3)	8 (15.4)	0.963 (0.320-2.896)	0.946
BDNF rs988748	CC	15 (28.8)	9 (16.4)	1.00 (Reference)	
	GG	11 (21.2)	14 (25.5)	0.437 (0.133-1.436)	0.173
	CG	26 (50.0)	32 (58.2)	0.443 (0.159-1.231)	0.118
	CG+GG vs. CC	37 (71.2)	46 (83.6)	0.441 (0.166-1.175)	0.101
	CG+CC vs. GG	41 (78.8)	41 (74.5)	0.786 (0.312-1.979)	0.609

*Adjusted for gender, age, education level, and APOE-4 allele carrier status; Bold values indicate P < 0.05.

### 3.4 BDNF polymorphism and BDNF levels

Covariance analysis was used to explore the serum BDNF levels of BDNF genotype polymorphisms in the elderly adults, as shown in **Table 4**. The results showed that in the healthy control group, there were no significant differences in BDNF levels among different genotypes at each locus. In the MCI group, carriers of the rs7103411 T allele had lower BDNF levels compared to CC homozygotes (CT+TT vs. CC) (*P* = 0.045). For rs6265, the AG genotype had significantly lower BDNF levels compared to the GG genotype (*P* = 0.028), and carriers of the G allele had significantly lower levels compared to A homozygotes (AG+AA vs. GG) (*P* = 0.026). For rs11030104, the AG genotype had significantly lower BDNF levels compared to the AA genotype (*P* = 0.049), and carriers of the G allele had significantly lower levels compared to A homozygotes (AG+GG vs. AA) (*P* = 0.045). For rs988748, carriers of the C allele had significantly lower BDNF levels compared to G homozygotes (CG+CC vs. GG) (*P* = 0.045).

**Table 4:** Relationship between BDNF rs7103411 polymorphism and BDNF levels.

Gene Locus	Genotyping	MCI		HC	
		BDNF (ng/ml),mean (SE)	*P*	BDNF (ng/ml),mean (SE)	*P*
BDNF rs7103411	TT (Reference)	9.338 (0.293)		9.740 (0.582)	
	CC	10.551 (0.364)	0.104	10.761 (0.477)	0.644
	CT	8.974 (0.271)	0.746	9.579 (0.249)	0.776
	CT+CC vs. TT	9.470 (0.208)	0.664	9.939 (0.226)	0.736
	CT+TT vs. CC	9.125 (0.199)	**0.045**	9.614 (0.228)	0.537
BDNF rs6265	GG (Reference)	10.512 (0.359)		10.409 (0.407)	
	AA	9.338 (0.306)	0.082	10.267 (0.457)	0.839
	AG	8.926 (0.284)	**0.028**	9.417 (0.277)	0.658
	AG+AA vs. GG	9.101 (0.207)	**0.026**	9.645 (0.234)	0.796
	AG+GG vs. AA	9.470 (0.208)	0.664	9.717 (0.235)	0.578
BDNF rs11030104	AA (Reference)	10.551 (0.362)		10.598 (0.382)	
	GG	9.390 (0.282)	0.105	9.371 (0.290)	0.754
	AG	8.917 (0.278)	**0.049**	8.909 (0.282)	0.811
	AG+GG vs. AA	9.125 (0.199)	**0.045**	9.810 (0.227)	0.766
	AG+AA vs. GG	9.390 (0.278)	0.654	9.994 (0.231)	0.823
BDNF rs11030100	GG (Reference)	9.349 (0.470)		10.292 (1.065)	
	TT	9.620 (0.199)	0.880	9.719 (0.244)	0.649
	GT	6.868 (0.639)	0.620	9.549 (0.812)	0.776
	GT+TT vs. GG	9.492 (0.207)	0.911	9.705 (0.231)	0.649
	GT+GG vs. TT	9.620 (0.205)	0.661	9.719 (0.241)	0.718
BDNF rs988748	CC (Reference)	9.053 (0.335)		9.740 (0.582)	
	GG	10.551 (0.363)	0.065	10.761 (0.477)	0.644
	CG	9.166 (0.253)	0.790	9.579 (0.249)	0.776
	CG+GG vs. CC	9.626 (0.387)	0.360	9.939 (0.226)	0.975
	CG+CC vs. GG	9.125 (0.199)	**0.045**	9.614 (0.228)	0.350

*Adjusted for gender, age, education level, APOE-4 allele carrier status, and BMI; Bold values indicate P < 0.05.

### 3.5 BDNF rs7103411 polymorphism interaction on cognitive function

After adjusting for gender, age, educational level, and APOE-4 allele carriage, multivariate logistic regression analysis showed a significant interaction between BDNF rs7103411 and social activity (*P* = 0.033). Further analysis revealed that individuals with the CT or TT genotype in rs7103411 polymorphisms of BDNF gene who participated to social activities showed a significantly reduced risk for MCI, as compared to individuals who did not participate to social activities(*OR* = 0.320, 95% *CI*: 0.117-0.878, *P* = 0.027), as shown in **Table 5**. No statistically significant differences were found at other genetic loci. (all *P >*0.05).

**Table 5:** Interaction between BDNF polymorphism and social activity.

SNP	Genotyping		Social Activity	*OR (95% CI)*	*P*
rs7103411	CT+CC vs. TT	CT+CC	Do Not Participate	1.000 (Reference)	
			Participate	3.477 (0.591-24.060)	0.179
		TT	Do Not Participate	1.378 (0.305-6.372)	0.675
			Participate	0.513 (0.121-2.162)	0.355
	CT+TT vs. CC	CT+TT	Do Not Participate	1.000 (Reference)	
			Participate	0.320 (0.117-0.878)	**0.027**
		CC	Do Not Participate	0.670 (0.150-3.000)	0.601
			Participate	2.488 (0.527-11.753)	0.250
	C allele vs. T allele	T	Do Not Participate	1.000 (Reference)	
			Participate	0.424 (0.155 - 1.123)	0.087
		C	Do Not Participate	1.031 (0.336 - 3.171)	0.957
			Participate	0.756 (0.281 - 1.988)	0.573
rs6265	AG+GG vs. AA	AG+GG	Do Not Participate	1.000 (Reference)	
			Participate	0.927 (0.33 - 2.554)	0.883
		AA	Do Not Participate	3.621 (0.661 -28.892)	0.165
			Participate	0.387 (0.092 - 1.473)	0.175
	AG+AA vs. GG	AG+AA	Do Not Participate	1.000 (Reference)	
			Participate	2.116 (0.393 - 12.07)	0.384
		GG	Do Not Participate	1.637 (0.356 - 7.779)	0.525
			Participate	0.571 (0.134 - 2.416)	0.439
	A allele vs. G allele	G	Do Not Participate	1.000 (Reference)	
			Participate	0.750 (0.275 - 2.009)	0.569
		A	Do Not Participate	1.563 (0.501 - 4.985)	0.443
			Participate	0.451 (0.162 - 1.220)	0.120
rs11030104	AG+GG vs. AA	AG+GG	Do Not Participate	1.000 (Reference)	
			Participate	0.197 (0.027 - 1.165)	0.084
		AA	Do Not Participate	0.465 (0.079 - 2.310)	0.362
			Participate	0.397 (0.073 - 1.763)	0.242
	AG+AA vs. GG	AG+AA	Do Not Participate	1.000 (Reference)	
			Participate	0.399 (0.143 - 1.077)	0.073
		GG	Do Not Participate	0.881 (0.288 - 2.68)	0.823
			Participate	0.701 (0.261 - 1.831)	0.472
	A allele vs. G allele	G	Do Not Participate	1.000 (Reference)	
			Participate	2.832 (0.516 -17.126)	0.236
		A	Do Not Participate	2.189 (0.331 - 16.46)	0.423
			Participate	0.446 (0.079 - 2.381)	0.345
rs988748	CG+GG vs. CC	CG+GG	Do Not Participate	1.000 (Reference)	
			Participate	0.879 (0.323 - 2.35)	0.798
		CC	Do Not Participate	2.142 (0.431-12.645)	0.365
			Participate	0.389 (0.095 - 1.445)	0.169
	CG+CC vs. GG	CG+CC	Do Not Participate	1.000 (Reference)	
			Participate	0.182 (0.026 - 1.053)	0.067
		GG	Do Not Participate	0.467 (0.079 - 2.322)	0.365
			Participate	0.410 (0.075 - 1.827)	0.260
	C allele vs. G allele	C	Do Not Participate	1.000 (Reference)	
			Participate	1.185 (0.446 - 3.194)	0.734
		G	Do Not Participate	1.625 (0.615 - 4.441)	0.332
			Participate	0.607 (0.275 - 1.324)	0.212

*Adjusted for gender, age, education level, and APOE-4 allele carrier status; Bold values indicate P < 0.05.

## 4 Discussion

Gene-environment interactions play a crucial role in complex diseases such as neurodegenerative disorders (38). We found a significant interaction between BDNF rs7103411 polymorphism and social activity. Among carriers of the T allele, those who engaged in social activities had a significantly lower risk of developing MCI compared to those who did not participate. Social activities, as a key lifestyle factor, have profound impacts on cognitive function and psychological health in the elderly adults. Especially in elderly populations, promoting social engagement and reducing feelings of loneliness may help maintain and enhance cognitive functions (31, 39, 40). Cognitive reserve might be a potential mechanism by which social isolation affects cognitive function (40), where having extensive social connections and engaging in challenging, complex social interactions can provide mental stimulation and thus build cognitive reserve (41). Ward et al.’s study revealed that the Val66Met polymorphism of the BDNF gene might indirectly influence cognitive function by regulating an individual’s use of cognitive reserves, particularly in executive functions (42). Additionally, elderly adults with depression or anxiety might experience more negative social interactions, which could exacerbate feelings of loneliness and isolation. Evans et al. highlighted the relationship between social isolation and symptoms of depression and anxiety, as well as their interaction with cognitive function in the elderly adults (43). A study has been shown that patients with early-onset AD who carry the ApoE4 allele in relatively early stages might develop a greater impairment on episodic verbal memory task, as compared to ApoE4 non-carriers (44). Furthermore, studies have revealed the interaction between BDNF and feelings of loneliness impacting cognitive decline, particularly manifesting as more pronounced semantic memory deterioration and increased feelings of loneliness among participants with lower BDNF expression levels (45). This finding further supports the protective mechanism of BDNF on cognitive functions.These findings, together with our study, emphasize the complexity of interactions among social engagement, gene polymorphism, and cognitive reserve, providing insights into the etiology and prevention strategies for MCI.

BDNF gene polymorphisms are a focal point in research on Alzheimer’s disease (AD) and Mild Cognitive Impairment (MCI), revealing their significant role as prognostic markers in memory decline and hippocampal atrophy in MCI patients, and their close association with the progression of MCI (15). In healthy adults with high levels of Aβ accumulation in the brain, carriers of the Met allele exhibit greater declines in cognitive function and hippocampal volume (20), further underscoring the potential role of the BDNF gene in regulating cognitive processes. This study found that BDNF rs7103411 polymorphism is associated with MCI risk. In a study of an elderly adults German cohort, rs7103411 polymorphism was significantly related to memory performance, with carriers of the C allele showing an increased risk of cognitive decline (including memory and perceptual speed) (22). However, in our research, individuals carrying the C allele were found to have a reduced risk of MCI compared to TT individuals. This could be due to differences in genetic background, gender stratification, and differences in cognitive function tests. A study in an English community of elderly adults did not find an association between rs7103411 and cognitive function, which might primarily be due to the use of a haplotype analysis method (25). Additionally, we observed lower serum BDNF levels in rs7103411 T allele carriers compared to CC individuals among MCI patients, further suggesting that BDNF gene polymorphism might affect cognitive functions and their decline by modulating BDNF expression levels. Moreover, cognitive function and susceptibility to MCI may be influenced by complex interactions among multiple genes (26). The effects of a single gene polymorphism may be offset or enhanced by the actions of other genes, making it difficult to consistently observe the impacts of individual polymorphisms across all studies (46).

This study did not find an association between polymorphisms rs6265, rs11030104, rs11030100, and rs988748 with susceptibility to Mild Cognitive Impairment (MCI). Beyond the limitations of sample size and study type, the cohort selected from community-dwelling elderly adults with MCI had relatively short disease duration and milder disease severity, which may have hindered the observation of correlations between cognitive abilities and both BDNF levels and genetic polymorphisms. Similarly, previous research has shown that rs6265 polymorphism is not significantly associated with the age of onset, severity, or rate of cognitive decline in Alzheimer’s disease (AD) (47, 48). No significant association was found between rs11030104 and cognitive decline in AD patients either (49). Currently, the understanding of the specific roles of the BDNF gene in cognitive function and the development of MCI is still limited. The biological effects of polymorphisms may involve complex molecular pathways and networks that are not yet fully understood and should be further explored in future research. For example, genetic variants affecting amyloid precursor protein (APP), tau binding proteins, immunity, and lipid metabolism play a critical role in AD, as shown by a largely cited study (50), with underwent an Author correction (51). Differences in definitions of MCI or cognitive decline and variations in studies across different subtypes may lead to contradictory conclusions (1, 52). Additionally, due to the limitations of the survey content, the impact of gene-environment interactions may not have been fully considered, which can also contribute to divergent study results (27, 53, 54).

This study indicates that multiple single nucleotide polymorphisms (SNPs) within the BDNF gene, including rs6265, rs11030104, rs7103411, and rs988748, are associated with serum BDNF levels in patients with Mild Cognitive Impairment (MCI). Reduced BDNF levels and signaling are linked to neurodegenerative diseases such as MCI, Alzheimer’s Disease (AD), and Parkinson’s Disease (PD) (3). Notably, the rs6265 polymorphism, consistent with findings from numerous studies, has been shown to correlate with BDNF levels and is closely associated with key neurophysiological processes involved in cognition (23, 55, 56). Specifically, rs6265 is linked to changes in hippocampal volume, cortical thickness, and abnormalities in synaptic connections, which are critical indicators of cognitive function (55, 56). Further research has also highlighted the role of rs6265 in learning, memory retention, executive functions, and the decline in perceptual speed among the elderly adults (57, 58). The evidence from this study further supports the significant role of the rs6265 polymorphism in cognitive dysfunction. Polymorphisms rs11030104, rs7103411, and rs988748 show differences in BDNF expression levels within haplotypes (59). Higher BDNF expression levels may be associated with poorer cognitive performance, including overall cognition, episodic memory, executive functions, visuospatial abilities, and semantic processing (60). Although our study identified differences in serum BDNF levels across multiple BDNF SNPs, aside from rs7103411, no other polymorphisms were found to be associated with MCI. This analysis might be influenced by the study’s sample originating from community-dwelling elderly adults individuals selected without self-perceived symptoms, whose symptoms may not be readily noticeable. Secondly, changes in serum BDNF levels as early biomarkers for MCI or AD might occur before the observable progression of the disease.

Current research on the peripheral BDNF levels in patients with AD and MCI shows inconsistent results. Some studies suggest that BDNF levels are elevated in MCI patients, possibly reflecting a compensatory neurorestorative mechanism at the early stages of AD (11, 13, 61). However, in AD patients, extensive neuronal damage and depleted cognitive reserves lead to a significant decrease in BDNF levels (4, 13). In rat models, homozygous rats for rs6265 show a notable reduction in BDNF release compared to heterozygous rats, yet they exhibit significantly enhanced synaptic growth capabilities (62). Some researchers hypothesize that a decrease in serum BDNF levels is a late event in the progression of AD hence changes in BDNF levels during the MCI stage might not be pronounced (4). Concurrently, our study observed a negative correlation between BDNF levels and scores on MMSE and orientation tasks in MCI patients. These findings further support the hypothesis that BDNF serves as a compensatory and protective mechanism in patients with MCI or early-stage AD. The role of BDNF in neuroprotection and neuroregeneration has been extensively studied, and our results did not find a significant correlation between serum BDNF levels and MCI status. This may be due to the influence of various biological, methodological, and environmental factors on serum BDNF levels.

Our study has certain limitations, including a small sample size, a cross-sectional study design, and potential confounding factors. Future research should employ larger sample sizes and longitudinal study designs, and consider a wider range of genetic and environmental factors to delve deeper into the mechanisms of BDNF in the development of MCI. Additionally, exploring the interactions between BDNF and other biomarkers will be an important direction for future studies. Previous research has primarily focused on the Val66Met polymorphism, with the relatively limited investigation into other polymorphic sites within the BDNF gene. This finding suggests that polymorphic sites other than Val66Met may also play roles in cognitive decline and susceptibility to MCI, which warrants further verification in future studies. Considering BDNF’s critical role in neuroprotection and neuroplasticity, our findings underscore the need for future research to thoroughly explore the complex biological mechanisms between BDNF and MCI, as well as the potential long-term effects on cognitive function in the elderly adults.

## Data Availability

The raw data supporting the conclusions of this article will be made available by the authors, without undue reservation.
